# Unveiling the Synergistic Potential: Bispecific Antibodies in Conjunction with Chemotherapy for Advanced Non-Small-Cell Lung Cancer Treatment

**DOI:** 10.3390/curroncol32040206

**Published:** 2025-03-31

**Authors:** Saqib Raza Khan, Daniel Breadner

**Affiliations:** 1 Division of Medical Oncology, Department of Oncology, Schulich School of Medicine & Dentistry, Western University, London, ON N6A 3K7, Canada; saqib.khan@lhsc.on.ca; 2 Verspeeten Family Cancer Centre, London Health Sciences Centre, London, ON N6A 5W9, Canada

**Keywords:** lung cancer, bispecific antibodies, chemotherapy, tumor microenvironment

## Abstract

Lung cancer remains the leading cause of cancer-related mortality worldwide, with non-small-cell lung cancer (NSCLC) accounting for the majority of the cases. Despite advancements in targeted therapies and immunotherapies, many patients still rely on chemotherapy, highlighting the need for innovative treatment strategies. Bispecific antibodies (bsAbs), which feature two distinct binding sites capable of targeting different antigens, have emerged as a promising therapeutic approach, particularly in combination with chemotherapy. This review explores the scientific evolution and clinical application of bsAbs in NSCLC, focusing on their synergistic potential with chemotherapy. BsAbs, such as amivantamab, which targets EGFR and MET, have demonstrated significant efficacy in clinical trials, particularly in patients with EGFR mutations. The combination of bsAbs with chemotherapy enhances immune-mediated tumor destruction by modulating the tumor microenvironment and overcoming resistance mechanisms. Recent clinical trials have shown improved progression-free survival and overall survival when bsAbs such as amivantamab are combined with chemotherapy, underscoring their potential to transform NSCLC treatment. Many other clinical trials are underway that are evaluating newer bsAbs, such as ivonescimab, which targets PD1 and VEGF. This review also discusses ongoing clinical trials investigating various bsAbs targeting EGFR, PD-1, PD-L1, HER2, and other pathways, highlighting the future directions of bsAb-based therapies. As the field evolves, bsAbs are poised to become a cornerstone of multimodal NSCLC treatment, offering more effective and personalized therapeutic options for patients with advanced disease.

## 1. Introduction

Lung cancer is the second most commonly diagnosed cancer and is the leading cause of mortality worldwide, with an estimated 1.8 million deaths as per the GLOBOCAN cancer statistics 2022 [1,2]. Non-small-cell lung cancer (NSCLC) accounts for more than 80% of the cases, with the majority (70%) presented in the advanced stage (stage III/IV) at the time of diagnosis [3]. Furthermore, targetable gene mutations that can act as therapy targets are present in less than 50% of the cases [4], with the rest of the patients ending up receiving either chemotherapy, immunotherapy, or chemo-immunotherapy as standard first-line (1L) palliative treatment [5,6,7]. Despite these efforts, the 5-year net survival rate in the advanced stage range is approximately 5–10% [7,8,9,10,11].

In NSCLC, the introduction of monoclonal antibodies has significantly improved the response rates and survival outcomes, leading to a growing interest in antibody-based therapies [10]. This interest has paved the way for developing bispecific antibodies (bsAbs), which feature two distinct binding sites capable of targeting different antigens [10]. Bispecific antibodies are among the fastest-growing drug classes in oncological clinical trials. As targeted therapy becomes increasingly precise, treatment paradigms are evolving, particularly for patients with advanced NSCLC. This patient cohort has experienced considerable shifts in the standard of care, driven by a deeper understanding of tumor resistance mechanisms and mutations that can be specifically targeted by therapy. Current guidelines from the National Comprehensive Cancer Network (NCCN) and Cancer Care Ontario (CCO) recommend testing for mutations, including EGFR, ALK, KRAS, ROS1, RET, BRAF, NTRK1/2/3, METex14 skipping, and HER2/ERBB2 [12,13]. By conducting an extended molecular panel via next-generation sequencing (NGS) before starting systemic treatment, physicians are better equipped to make informed decisions for personalized treatment plans tailored to each patient.

The clinical significance of bispecific antibodies gained prominence following the initial approval of blinatumomab by the U.S. Food and Drug Administration (FDA) for treating acute lymphoblastic leukemia (ALL) [14]. Amivantamab, a bispecific antibody targeting both EGFR and MET, has since received multiple approvals for use in NSCLC. After demonstrating a 40% response rate in a phase I trial focused on patients with EGFR exon 20 insertion mutations [15], the sustained efficacy of amivantamab has been observed in subsequent trials, both as a first-line combination therapy for EGFR exon 20 insertion mutations [16] and in first and subsequent lines of treatment for EGFR exon 19 deletions and exon 21 L858R mutations [17,18]. These approvals by the FDA and European Medicines Agency (EMA) mark a significant advancement in the use of bsAbs for the treatment of NSCLC.

Bispecific antibodies can take on various forms and mechanisms of action, depending on the cell type and genetic engineering platform used in their development. This review article discusses the scientific evolution of bsAb use in NSCLC, its structure and mechanisms of action, clinical trials, the synergistic potential when combined with systemic chemotherapy, and the future directions in this field.

## 2. Bispecific Antibodies and the Science Behind Their Construction

**Figure 1:**
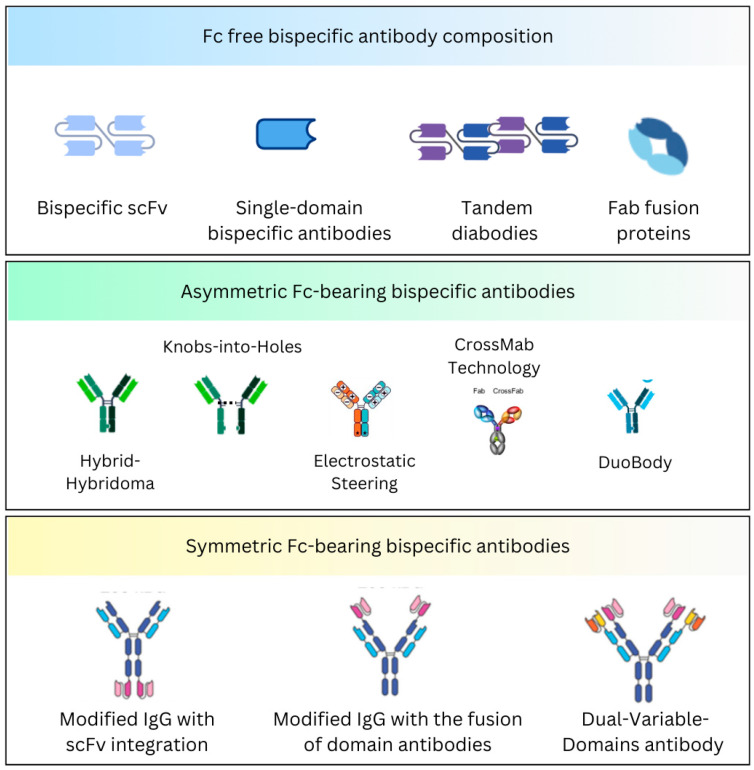
Briefly depicts the Fc-free and Fc-bearing BsAb composition.

Natural antibodies have two targeting arms but are monospecific and bind to the same target antigen. Bispecific antibodies identify different epitopes on the same or different antigens. The antigen-binding sites are developed by the variable domains of light (VL) and heavy (VH) chains; therefore, constructing bispecific molecules is difficult because bispecific antibodies require two different light chains and two different heavy chains. Various strategies have been developed to establish a correct assembly. A significant distinction is the presence or absence of the Fc region (Figure 1).

### 2.1. Fc-Free Bispecific Antibody Composition

These are non-IgG-based antibodies that deliver weak immunogenicity. Because they lack an Fc portion, these formats achieve higher tissue penetration but have relatively short half-lives. Various subtypes have been identified.

#### 2.1.1. The Bispecific Tandem Single-Chain Variable Fragments

The single-chain variable fragment (scFv), the most common derivative of the VH and VL domains, constitutes the minimal antigen-binding site of an antibody [19]. Bispecific antibodies with the single-chain configuration are connected via a linker or a connector. Several linkers have been identified and utilized, such as hydrophilic linkers, short alanine linkers, and those that are derived from multiple immunoglobulin and non-immunoglobulin molecules [20,21].

Moreover, this format forms the bispecific T-cell engager (BiTE) molecules [22,23]. The binding domain consists of two scFv chains from a monoclonal antibody that binds to tumor antigens and CD3 on T-cells, respectively, and are linked by a peptide chain connector [24]. Blinatumomab was the first BiTE molecule developed for relapsed/refractory Philadelphia-negative B-cell acute lymphoblastic leukemia (ALL) [25].

Bispecific killer cell engagers (BiKEs) were further developed via tandem scFvs, which includes the anti-CD16 scFv. In a colorectal cancer study, the anti-CD16 scFv was linked with a 20-amino acid connector derived from human muscle aldolase to an anti-CD 33 scFv [26].

#### 2.1.2. Single-Domain Bispecific Antibodies

Single-domain antibodies, such as VHH (variable domain of heavy-chain-only antibody) and VNAR (variable domain of new antigen receptor), can be used to make bispecific molecules [27]. These are smaller-molecular-weight antibodies with considerable efficacy in penetrating tissues. The VHH domains came from camelid antibodies, while the VNAR domains came from shark antibodies, and each was linked with a flexible sequence to enhance the stability [28,29]. These constructs can bind multiple targets at a time. Nanobodies are used in clinical trials. A preclinical study with a novel bispecific nanobody targeting CXCR and PDL1 revealed antitumor activity in pancreatic cancer cells [30].

#### 2.1.3. Tandem Diabodies and Derivatives

Bispecific diabodies have been engineered for various uses, including the redirection of effector cells, effector molecules, and other therapeutic applications [31,32,33,34]. These bivalent molecules contain two chains, each containing a VH and VL domain [35,36]. In the diabody structure, the variable domains are connected by a short linker. Using this format, efforts have been made to create trivalent and tetravalent bispecific molecules that can bind multiple sites on an antigen. An example is AFM13, a tandem diabody targeting CD30 on lymphoma cells and CD16a on NK cells, which has demonstrated efficacy in Hodgkin lymphoma [37].

#### 2.1.4. Fab Fusion Proteins

Using Fabs, which are heterodimeric molecules with light chain and heavy chain fragments (Fd), Fc-less bispecific molecules can be constructed. They can generate bivalent, bispecific molecules and trivalent, trispecific fusion proteins by adding scFvs to their ends [38,39,40]. These fusion proteins are stable and functional in bispecific binding and display low aggregation tendencies under physiologic conditions. The flexibility of the molecules is modulated by the connectors between the Fab fragments and scFvs. Multiple designs have been developed, such as TriFabs, trivalent bispecific molecules, and Fab-Fab fusion proteins, which target multiple antigens, including EGFR, Her-2, CD19, and CD16 [41,42,43]. These formats can enhance the therapeutic targeting, including immune retargeting via T-cell receptors (TCRs) [44].

#### 2.1.5. Other Miniantibodies

Heterodimerizing peptides (miniantibodies), such as leucine zippers from proteins like Jun and Fos, can be used to construct Fc-less bispecific antibodies [45,46]. These peptide molecules facilitate the fusion of Fabs or scFvs to form bispecific heterodimers or tetravalent fusion proteins. The CH1 and CL domains can also be used to develop stable bispecific miniantibodies targeting antigens like CD16 and CEA [47].

Other non-immunoglobulin methods, such as the “dock-and-lock (DNL)” technique, allow for the congregation of trivalent or hexavalent molecules [48]. Using proteins like albumin is another approach to enhance the half-life and effectiveness of bispecific antibodies [49].

### 2.2. Fc-Bearing Bispecific Antibody Composition

These are IgG-based antibodies with an Fc region. They have prolonged half-lives due to the recycling of the Fc region and have the propensity to engage immune effector functions, such as the complement-dependent cytotoxicity (CDC) and antibody-dependent cellular cytotoxicity (ADCC) [46]. These antibodies can be mapped into symmetric and asymmetric configurations. Compared to the symmetrical bispecific antibodies, which maintain a balanced structure and are easier to produce, the asymmetrical formats are complex to engineer but offer greater flexibility to target different antigens [46]. The design choice depends upon the therapeutic goal, with asymmetrical schemes often favored for more precise or multi-targeted treatments.

#### 2.2.1. Asymmetric Fc-Bearing Bispecific Antibodies

**Hybrid-Hybridoma**

These bispecific antibodies combine heavy and light chains from two different hybridoma cells to produce monoclonal antibodies [50]. The product is an asymmetric bispecific antibody that can also come from different species, such as mice and rats, to form triomabs [51]. Catumaxomab is a bispecific antibody that targets EpCAM and CD3. It is constructed in this format and consists of IgG2a and IgG2b from mice and rats, respectively [51].

**Knobs-into-Holes**

This technique involves genetically engineering the CH3 domains of the Fc region. One of the heavy chain amino acids is modified to create a Knob, and the other forms a Hole, warranting selective heterodimerization [52,53]. Compared to the Hybrid-Hybridoma technique, this approach is highly effective in constructing bispecific antibodies with minimal chain mispairing.

**Electrostatic Steering**

Electrostatic steering introduces charge pairs into the CH3 domains or hinge region to boost heterodimerization and circumvent homodimer formation [54]. This strategy uses charged amino acids to establish the desired heterodimer flock together. This method has generated bispecific antibodies against Her-2 and EGFR [55]. Furthermore, it is often combined with other techniques like Knobs-into-Holes to augment the overall effects.

**CrossMab Technology**

The CrossMab strategy is unique, as it swaps specific domains between the light and heavy chains to ensure the correct pairing of chains and the construction of bispecific antibodies. The fragments can be exchanged at the level of the Fab (CrossMab Fab), variable domains (CrossMab VH-VL), and invariant domains (CrossMab CH1-CL) [56,57,58].

**Post-Assembly Methods**

In some instances, bispecific antibodies are generated by expressing two half-antibodies separately. These half-antibodies are then fortified to form the complete bispecific molecules [59]. This approach can be more complex but allows for greater control over the assembly process.

**DuoBody**

DuoBody is a bispecific antibody platform technology developed to construct molecules that can concomitantly bind to two different targets. This highly flexible platform works by exchanging two different monoclonal antibodies. Epcortimab (GEN3013) is a DuoBody that targets CD20 on B-cells and CD3 on T-cells, and which is being investigated in relapsed/refractory B-cell lymphoma [60]. Amivantamab is another bispecific antibody that targets EGFR and MET, and it is designed to treat NSCLC patients with EGFR exon 20 insertion mutation-positive [15].

#### 2.2.2. Symmetric Fc-Bearing Bispecific Antibodies

**Modified IgG with scFv integration**

This method involves integrating single-chain variable fragments to the C-terminus or N-terminus of the heavy or light chain, resulting in a symmetric, tetravalent structure [61]. These molecules maintain two binding sites for each antigen and are highly stable, with preserved Fc effector function and a half-life similar to the parental IgG [46,62].

**Modified IgG with the fusion of domain antibodies**

Single-variable domain (sVD) antibodies have been employed to produce modified bispecific molecules. Shen J et al. proposed that an sVD targeting PDGFRα was linked via a connector to the N-terminus of the light chain of the anti-VGFR2 IgG, maintaining both the antibodies’ antigen-binding and neutralization activity [63].

**Dual-Variable-Domains antibody (DVD-Ig)**

This format features additional heavy and light variable domains compounded with IgG heavy and light chains, forming a tetravalent IgG-like molecule with an Fc region. This format has been applied to create diverse molecules, including EGFR and HER3 [64], CEA and DOTA [65], CD20 and CD47 [66], and CD20 and HLA-DR [67].

**Others**

CrossMab technology can be applied to symmetric formats using a second Fab fused via a linker to the C-terminus of an IgG molecule. DARTs (dual-affinity retargeting molecules) enhance diabodies by stabilizing the scFv through additional disulfide bonds, constituting a more stable and potent format [68].

In vitro and animal models have demonstrated increased tumor cell killing with bsAbs. Xenograft models suggest superior efficacy in combination with chemotherapy. While preclinical models show enhanced activity, real-world factors such as patient heterogeneity, toxicity, and resistance mechanisms impact clinical outcomes.

## 3. Mechanism of Action of Bispecific Antibodies

**Figure 2:**
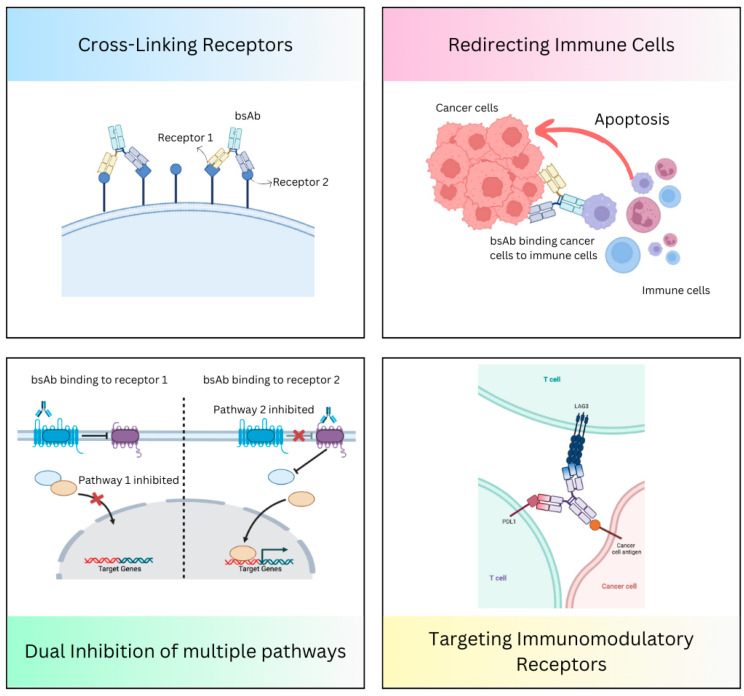
Mechanism of action of bispecific antibodies.

Bispecific antibodies that concomitantly connect two different antigens or epitopes have several unique action mechanisms. This diverse mechanism of action (MOA) makes them highly adaptable in therapeutic applications, with an increased potential to work synergistically. Some of the significant MOAs of bispecific antibodies include the following (Figure 2).

### 3.1. Redirecting Immune Cells to Tumor Cells

Bispecific antibodies can attach to immune effector cells, such as NK cells or T-cells, and then direct them to tumor cells by binding both to immune cell receptors (e.g., CD3 on T-cells) and tumor-associated antigens (e.g., CD19 on B-cells or DLL3 on neuroendocrine tumors). A bispecific T-cell engager (BiTE) molecule involves this MOA [22,23].

### 3.2. Dual Inhibition of Downstream or Underlying Signaling Pathways

Bispecific antibodies can bind to two different signaling pathways simultaneously, driving tumor growth by targeting two receptors on the tumor cells and preventing tumor escape. Amivantamab targets EGFR and MET, and it is designed to treat NSCLC patients with EGFR mutation-positive [15].

### 3.3. Cross-Linking of the Receptors

Bispecific antibodies can work by cross-linking two receptors on the tumor surface, causing dimerization and inhibiting the receptor signaling. Zenocutuzumab is a bispecific antibody carrying two different Fab arms directed against Her2 and Her3 [69].

### 3.4. Targeting Multiple Immunomodulatory Receptors

Bispecific antibodies can bind to different targets on T-cells [or tumor-infiltrating lymphocytes (TILs)] and modulate immune responses. FS118 is a bispecific antibody that targets LAG3 and PDL1 [70]. Cadonilimab (AK104) targets PD-1 and CTLA-4, and works by simultaneously blocking these immune checkpoints, enhancing T-cell activation and immune response against tumor cells [71].

## 4. Bispecific Antibodies Approved by the FDA in Oncology

**Figure 3:**
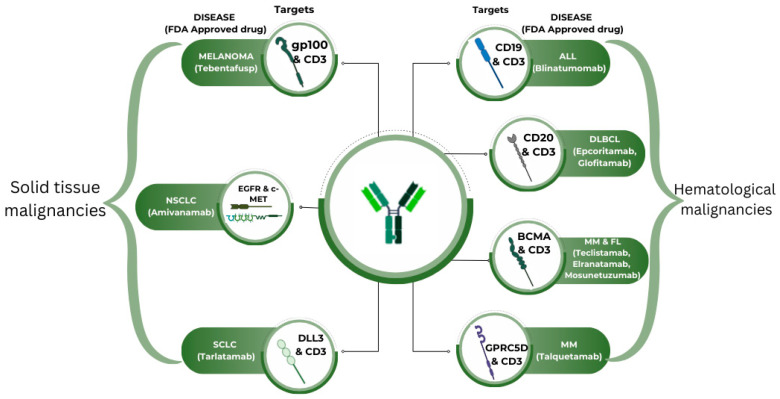
FDA-approved bispecific antibodies in oncology.

As of August 2024, the Food and Drug Administration (FDA) has approved twelve bispecific antibodies in different settings. Ten of these have been approved for oncology practice (Figure 3). Table 1 briefly overviews the FDA-approved bispecific antibodies in oncology. Many bispecific antibodies are in the development phase and need approval.

## 5. The Evolution of Bispecific Antibodies in NSCLC Treatment

Over the past decades, the treatment of NSCLC has undergone a profound transformation, driven by the advancement in modern biotechnology. Among the most noticeable innovations is the emergence of bispecific antibodies which seamlessly integrate precision targeting with immune activation. Currently, amivantamab-vmjw is the only FDA-approved bispecific antibody available for treating advanced NSCLC in EGFR mutation-positive disease. By targeting both EGFR and MET, amivantamab-vmjw overcomes the resistance mechanisms associated with EGFR mutation and MET amplifications. After binding to these targets, it allows for receptor degradation, blocking the downstream signaling pathways that endorse cancer cell division and survival. Moreover, augmenting ADCC further engages immune effector cells, leading to tumor cell killing.

**Figure 4:**
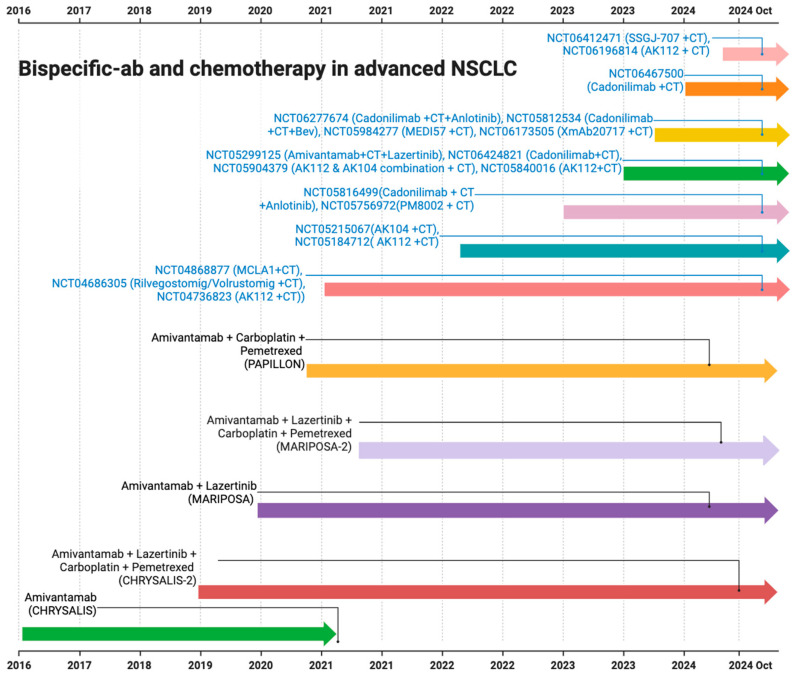
The timeline representing various clinical trials on bispecific antibodies with chemotherapy in NSCLC (AK112 is ivonescimab and AK104 is cadonilimab).

The FDA initially approved amivantamab in May 2021 (Figure 4). The approval was based on phase I CHRYSALIS trial results, which included 81 platinum-refractory patients with EGFR exon 20 insertion mutation-positive. The study showed an ORR of 40% (95% CI, 29 to 51), with an mDOR of 11.1 months (95% CI, 6.9 to not reached) [15]. Following this, the combination of amivantamab with other tyrosine kinase inhibitors (TKIs) and/or chemotherapy was evaluated, and many other trials are in the development phase. CHRYSALIS-2 evaluated lazertinib with amivantamab in different cohorts [81]. Cohort A, which included EGFR exon 19 deletion (ex19del)/L858R mutation-positive heavily pretreated platinum-refractory patients, including those who previously progressed on osimertinib, revealed an ORR of 36% (95% CI, 23–51) [82]. Interestingly, cohort C evaluated amivantamab and lazertinib in atypical EGFR mutation-positive (e.g., S768I, L861Q, and G719X), excluding EGFR exon 20 insertion mutation-positive, NSCLC patients, and showed an ORR of 55% (95% CI, 40–69) and 45% (95% CI, 29–62) in treatment-naive and refractory NSCLC patients, respectively [83].

The phase III MARIPOSA trial also led to the approval of amivantamab and lazertinib in patients with EGFR ex19delor L858R substitution mutation in advanced treatment-naive NSCLC. Amivantamab and lazertinib showed a median PFS of 23.7 months (95% CI, 19.1 and 27.7) compared to the 16.6 months (95% CI, 14.8 and 18.5) with the osimertinib monotherapy [84]. The updated data were presented at the World Conference of Lung Cancer (WCLC)-2024. At a median follow-up of 31.1 months, the combination led to a median OS that was not achieved (NE; 95% CI, NE-NE) versus 37.3 months (95% CI, 32.5-NE) with osimertinib [85]. Subsequent studies also explored the role of bispecific antibodies in combination with chemotherapy (Figure 4). PALOMA3 is currently evaluating the subcutaneous formulation of amivantamab and comparing it with intravenous formulations in combination with lazertinib to reduce the dose times [86].

## 6. Potential Synergism of Bispecific Antibodies and Chemotherapy in NSCLC

The potential synergistic effects of bispecific antibodies and cytotoxic chemotherapy in managing NSCLC arise from the different mechanisms of action of both therapies. The synergistic relationship primarily involves the propensity of chemotherapy to create an environment in which the bispecific antibodies can more effectively target cancer cells, strengthen immune-mediated tumor destruction, and overcome resistance mechanisms.

Among the 1L treatment options for advanced-stage NSCLC, platinum-based systemic chemotherapy forms the backbone. Platinum agents produce its antitumor effect by disrupting cellular pathways in tumor cell survival. Establishing cross-links in the DNA inhibits DNA replication and transcription, eventually triggering apoptosis and programmed cell death [87]. On the other hand, microtubular stabilization by enhancing the action of tubular dimers or disrupting the folate-dependent metabolic process crucial for DNA synthesis are some of the other commonly used preferred agent’s MOAs, leading to cell cycle inhibition [88,89]. Due to the cytotoxic effects, the cancer cells may upregulate stress signals and death receptor pathways in this weakened state, making them more assailable to immune-mediated killing.

Moreover, chemotherapy can modify the tumor microenvironment (TME) by consuming suppressive immune cells, such as myeloid-derived suppressor cells (MDSCs) and regulatory T-cells (Tregs), which, in normal circumstances, inhibit the antitumor immune response [90]. Additionally, the EGFR-targeted drugs lower the apoptotic threshold [91]. Consequently, this effect minimizes the tumor’s immune evasion magnitude, creating a more permissive environment for immune attack. In this more susceptible TME, bispecific antibodies that overpass tumor cells with T-cells (such as CD3-targeting bispecific antibodies) or NK cells (through Fc receptor engagement) can instigate robust immune activation and tumor cell death.

In the PAPILLON study, amivantamab was combined with carboplatin and pemetrexed (CP) to treat patients with advanced NSCLC [16]. The chemotherapy weakened the tumor and modulated the TME, leading to increased tumor antigen exposure. Amivantamab acted by blocking the vital oncogenic pathways regulated by MET and EGFR, further recruiting immune cells to the tumor and enhancing tumor killing through mechanisms like ADCC. Hence, the study showed the superior efficacy of amivantamab in combination with chemotherapy compared to standard CP alone in the first-line setting for EGFR exon 20 insertion mutation-positive NSCLC. Compared to the CP arm, a statistically significant improvement in PFS was seen in the amivantamab plus CP arm with a hazard ratio of 0.40 (95% CI, 0.30 and 0.53; *p*-value < 0.0001). The median PFS was 6.7 months (95% CI, 5.6 and 7.3) in the chemotherapy-alone arm; however, it was significantly higher in the combination arm at 11.4 months (95% CI, 9.8 and 13.7), with an ORR of 73% [16]. Patient-relevant endpoints from the PAPILLON study also revealed that the emotional functioning, role functioning, cognitive functioning, as well as global health status in patients who received amivantamab and chemotherapy was higher than those received systemic chemotherapy alone [92].

Consistent results were also observed in the MARIPOSA-2 study in patients with EGFR exon 19 deletion, and in L858R mutation advanced NSCLC patients who progressed on or after osimertinib [17]. The combination of amivantamab with chemotherapy and amivantamab plus lazertinib with chemotherapy showed a higher ORR of 64% and 63%, respectively, compared to the chemotherapy alone (36%), with a significant *p*-value of <0.001. A statistically significant intracranial PFS of 12.5 and 12.8 versus 8.3 months in these groups was also seen, with HRs of 0.55 and 0.88 for intracranial disease progression or death, respectively [17]. Recently, a second interim analysis of MARIPOSA-2 data revealed a persistent median OS benefit of 17.7 months with amivantamab plus chemotherapy versus 15.3 months with chemotherapy alone (HR, 0.73; 95% CI, 0.54–0.99; *p* = 0.039) [93] (Figure 4).

Furthermore, a multicenter phase II study in China evaluated the role of ivonescimab (AK112/SMT112), a bispecific antibody that targets PD-1 and VEGF (vascular endothelial growth factor) [94]. HARMONI-A, phase III study, confirmed the efficacy and safety of ivonescimab in combination with chemotherapy in patients with NSCLC who failed prior EGFR TKIs. The PFS was significantly improved with ivonescimab plus chemotherapy (HR, 0.46 [0.34, 0.62]; *p* < 0.0001). Other phase II trials are underway [95]. KN046, a bispecific antibody targeting PD-L1 and CTLA4, also showed promising results in combination with systemic chemotherapy, with an ORR of 46% (95% CI, 35.2–57.0%) and a median DOR of 8.1 (95% CI, 4.14–13.90) months in a phase II study of advanced metastatic NSCLC [96]. This complementary activity of bispecific antibodies with chemotherapy in different settings for the treatment of NSCLC patients represents a unique approach in oncology practice, combining the strength of both therapies to augment the efficacy. Table 2 briefly overviews various ongoing clinical trials utilizing bispecific antibodies in conjunction with either single-agent or doublet chemotherapy with or without additional agents in advanced NSCLC.

Bispecific antibodies introduce a novel class of adverse events, some of which overlap with chemotherapy-related toxicities; however, others are unique due to their dual-targeting mechanism. The significant adverse effects include cytopenias, transaminitis, diarrhea, tumor lysis syndrome, infusion reactions, and dermatological and nail toxicities. Additionally, cytokine release syndrome (CRS), tumor flare, neurotoxicity, and immune effector cell-associated neurotoxicity syndrome or ICANS are rare but specific to bsAbs [97,98]. These toxicities can significantly impact a patient’s quality of life (QOL) by causing physical discomfort, emotional distress, functional limitations, and social withdrawal. Severe fatigue, infections due to cytopenias, and gastrointestinal issues can reduce daily activity levels, while painful skin and nail toxicities may interfere with routine tasks and self-care. Moreover, neurotoxicity, including cognitive impairment and confusion, can further impair independence and work productivity. The need for frequent hospital visits, prolonged monitoring, and supporting interventions adds to the treatment burden, leading to psychological distress and potential treatment discontinuation. Individual patient factors, such as pre-existing comorbidities and prior treatment history, can impact both the likelihood and severity of bsAb-associated toxicities. Personalized treatment approaches and close monitoring for potential adverse effects are crucial to reduce the risk of complications

## 7. Precision Targeting with Bispecific Antibodies in NSCLC

Several bispecific antibodies are currently in the development phase, and others are in the approval process for the treatment of NSCLC. Various targets have been identified for this disease cohort. These include the following.

### 7.1. EGFR-Targeted Bispecific Antibodies in NSCLC

#### 7.1.1. EGFR/cMET

EGFR (epidermal growth factor receptor) mutations account for roughly 15–20% of all NSCLC patients, while MET (mesenchymal–epithelial transition factor) exon 14 alteration is found in 3% [99,100]. Amivantamab, which targets EGFR/cMET, is the only approved bispecific antibody for the treatment of NSCLC patients [15].

HS20117 is another fully human EGFR/cMET-targeted bispecific antibody under evaluation in a clinical phase I study of NSCLC and other solid cancer patients [101].

MCLA-129, an anti-EGFR/cMET bispecific antibody, is under phase I clinical trial in combination with befotertinib for safety and tolerance in advanced NSCLC patients with EGFR-sensitive mutations [102]. A phase I/II study is also currently evaluating its role in osimertinib-resistant NSCLC patients in combination with chemotherapy [103].

EMB-01 (bafisontamab) is a bispecific EGFR/cMET-targeted antibody currently under evaluation in a first-in-human (FIH) phase I/II study in advanced neoplasms, including NSCLC [104]. The study will investigate the adverse events, ORR, and maximum tolerated dose.

#### 7.1.2. EGFRxCD28

REGN7075 is an EGFRxCD28 costimulatory bispecific antibody. A phase I/II study is actively investigating RENG7075 in combination with chemo-immunotherapy in advanced NSCLC patients as part of one study cohort. The study will evaluate the side effects of REGN7075 that may be experienced by the patients and its safety in combination with chemo-immunotherapy [105].

#### 7.1.3. EGFRx41BB

HLX35 is a recombinant human anti-EGFR and anti-41BB bispecific antibody. A phase I study is investigating its safety, tolerability, and pharmacokinetics in advanced solid cancers, including NSCLC patients [106].

#### 7.1.4. EGFR/HER3

SI-B001 is an EGFR/HER3 bispecific antibody. A phase I study is investigating its dose-limiting toxicity (DLT) and maximum tolerating dose (MTD) in advanced tumors, including NSCLC [107].

#### 7.1.5. EGFRxCD3

TAK-186, or MVC-101, is a COnditional Bispecific Redirected Activation (COBRA) protein that targets EGFR and CD3. An ongoing phase I/II study is evaluating its role in platinum-refractory OR post-TKI OR ICI-refractory advanced NSCLC patients. In addition to safety and tolerability, the study will measure outcomes, including the PFS, ORR, OS, and DOR [108].

### 7.2. PD-1 or PD-L1-Targeted Bispecific Antibodies in NSCLC

#### 7.2.1. PD-1/CTLA4 or PD-L1/CTLA4

AK104, also known as cadonilimab, is a tetravalent bispecific antibody that targets PD-1 and CTLA4. Multiple phase I and II clinical trials are investigating this bispecific antibody in NSCLC. In a phase II study of advanced NSCLC, investigators are looking at its safety and tolerability in combination with pemetrexed and anlotinib in T790m-negative TKI-resistant patients [109]. Interestingly, cadonilimab efficacy in untreated brain metastasis has also been investigated in a phase II clinical trial. The study focused on EGFR- and ALK-negative advanced NSCLC patients with untreated brain metastases, and considered combination treatment with cadonilimab + bevacizumab + platinum-based chemotherapy. The outcome measures include intracranial PFS, OS, DOR, and OS [110].

KN046 is a recombinant humanized PD-L1/CTLA4 bispecific antibody. A multicenter phase II study in China is investigating its antitumor activity in terms of the ORR, DOR, DCR, and OS in advanced EGFR- and ALK-negative treatment-naive and ICI refractory PD-L1-positive NSCLC patients [111].

Volrustomig (MEDI5752) is another novel bispecific antibody that targets PD1 and CTLA4. The phase III “eVOLVE-Lung02” is an ongoing study investigating its effectiveness in 1 L advanced PD-L1-positive NSCLC patients in combination with platinum-based systemic chemotherapy [112]. Similarly, Evolve-Meso is a global phase III study evaluating volrustomig in combination with chemotherapy in unresectable pleural mesothelioma [113].

SIB003, lorigerlimab (MGD019), and vudalimab (XmAb20717) are other bispecific antibodies that target PD-1/CTLA4, and which are under investigation in phase I and II studies in solid cancer malignancies, including NSCLC patients [114,115,116].

#### 7.2.2. PD-1/VEGF or PD-L1/VEGF

PM8002 is an anti-PD-L1/VEGF bispecific antibody. A phase II/III study in China is actively investigating its role in TKI-resistant nonsquamous NSCLC patients in combination with systemic chemotherapy. The study will evaluate the comparative efficacy of PM8002 in combination with carboplatin and pemetrexed versus placebo and systemic chemotherapy regarding the ORR, PFS, and OS [117].

Ivonescimab (AK112) is an anti-PD-1/VEGF bispecific antibody under investigation in various clinical trials for treating NSCLC alone or in combination with systemic chemotherapy [94]. In the phase II study, patients were stratified into the following three cohorts: (1) treatment-naïve individuals without EGFR/ALK driver mutations, (2) patients with EGFR-sensitive NSCLC who had experienced disease progression following prior targeted therapy, and (3) those who had failed prior PD-1/PD-L1 inhibitors and platinum-based chemotherapy. In combination with chemotherapy, ivonescimab demonstrated promising efficacy across all cohorts, yielding ORRs of 53.5% (95% CI, 36.9–67.1), 68.4% (95% CI, 43.4–87.4), and 40% (95% CI, 19.1–63.9), respectively. Furthermore, as previously discussed, the phase III HARMONI-A trial validated the efficacy and safety of ivonescimab in combination with chemotherapy in patients who had progressed on prior EGFR TKIs, with a significant improvement in the PFS [94,95,118,119].

SSGJ-707 is another anti-PD-1/VEGF bispecific antibody. A phase II study is recruiting advanced NSCLC patients in a 1 L setting to evaluate the safety, tolerability, and response rate assessment of SSGH-707 in combination treatment with systemic chemotherapy [120].

The majority of the other PD-1- or PDL-1-targeted bispecific antibodies are in early-phase clinical trials, currently under investigation, or, in some, showing antitumor activity according to the preliminary data. These are summarized in Table 3.

### 7.3. HER2-Targeted Bispecific Antibodies in NSCLC

#### 7.3.1. HER2/HER3

Zenocutuzumab (MCLA-128) is an anti-HER2/HER3 bispecific antibody. In a multicenter phase II study, participants in group 2 with documented NRG1 fusion-positive NSCLC were enrolled to receive IV MCLA-128 to assess its safety and tolerability, ORR, DOR, PFS, and OS. The study is estimated to enroll 250 patients and is expected to be completed in 2026 [121].

#### 7.3.2. Dual-Domain HER2

Zanidatamab (ZW25) is a bispecific antibody that targets two distinct extracellular domains of HER2 (ECD2 and ECD4). A phase I study is investigating its role in solid cancers, including NSCLC patients expressing HER2 [122]. The study is estimated to enroll 279 patients and is expected to be completed in 2024.

### 7.4. OTHERS

#### 7.4.1. ROR1/CD3

NVG-111 is a two-armed scFv-Fc format bispecific antibody that targets ROR1 receptors on cancer cells and CD3 antigens on immune cells. A phase I study in solid tumors is investigating its efficacy and safety, focusing initially on NSCLC patients [123].

#### 7.4.2. B7H3/CD28

XmAb808 is a fully humanized B7H3xCD28 bispecific antibody. It delivers CD28-medicated co-stimulation of T-cells at the interface of B7H3-expressing tumors. A first-in-human (FIH) study is actively investigating its potential efficacy, safety, response, and survival outcomes in solid cancers, including NSCLC patients [124].

#### 7.4.3. B7H4/CD3

GEN1047 is another bispecific antibody that targets B7H4 on the surface of the tumor cells and CD3 antigen on immune cells. Its efficacy and safety are currently under investigation in a phase I/II study on solid cancers, including squamous NSCLC patients [125].

#### 7.4.4. EpCAM/CD3

Solitomab (MT110 and AMG 110) is a BiTE antibody that targets the epithelial cell adhesion molecule (EpCAM) and CD3 on T-cells. Went P. et al. proposed in their study that there is a high expression of the EpCAM protein in lung cancer, as high as 65%, and that it has a negative correlation with survival [126]. In a multicenter phase I study, solitomab showed early signs of antitumor activity in patients with refractory solid cancers, including NSCLC, with most patients experiencing DLTs [127].

#### 7.4.5. DLL4/VEGF

ABT-165 (dilpacimab) is a dual variable domain immunoglobulin (DVD-Ig) bispecific antibody that targets delta-like ligand 4 (DLL4) and VEGF. The early phase of the phase I/Ib study showed antitumor activity in advanced solid tumors, with a 10.9% ORR and a median PFS of 3.7 months (95% CI, 2.7–3.9) [128]. Patient enrollments to cohorts C and D are still in process, as updated in Dec’23 [128,129].

#### 7.4.6. MET/MET

Davutamig (REGN5093) is an innovative bispecific antibody that targets two distinct epitopes of MET, inhibiting ligand binding and driving MET internalization and degradation. Clinical trials exploring this bispecific antibody are on their way. A phase I/II, first-in-human (FIH) study evaluated davutamig in subsequent-line settings in patients with MET-altered advanced NSCLC (exon 14 skipping mutation/overexpression/amplification) [130]. The ORR was 25% in MET-targeted naive patients with exon 14 skipping mutations and 13% in those with MET overexpression/amplification, with a manageable safety profile [130].

## 8. Perspectives and Conclusions

The use of bispecific antibodies in combination with systemic chemotherapy for advanced NSCLC carries substantial promise, while further studies are needed to appreciate the full breadth of their clinical implications. The data from the MARIPOSA2 and PAPILLON clinical trials has led to practice-changing proof of the synergistic potential of combining bispecific antibodies and cytotoxic therapy. The potential to augment and diversify these treatment combinations is enormous. Furthermore, optimizing patient selection to identify subgroup cohorts that may benefit from this therapeutic approach is an essential area to focus on. Investigating new biomarker expressions could further stratify patient groups and personalized treatment, intensifying the response rate and lowering the toxicity.

There is a strong rationale for investigating alternative bispecific antibody combinations. Multiple clinical trials have explored the safety and antitumor activity of different bispecific antibodies in NSCLC, targeting cellular pathways as a monotherapy. Using these bispecific antibodies that target the immunosuppressive pathways within the TME, such as CTLA4/LAG3 or PD-1/VEGF, in tandem with chemotherapy, could supplement the immune-mediated destruction of cancer cells, as seen in the HARMONI-A study following the previous failure of immunotherapy in EGFR-mutated NSCLC [95]. These combinations can target tumor growth pathways while also reducing immune suppression within the TME, possibly contributing to long-term survival in advanced NSCLC patients. Given the distinctive propensity of bispecific antibodies to recruit immune cells, combining them with different chemotherapeutic agents that surge tumor antigen presentation could further potentiate T-cell activity and immune cell participation against tumor cells.

Moreover, prospective clinical trials could also investigate different dosing regimens and the schedules of bispecific antibodies in combination with systemic chemotherapy. A sequential or staggered dosing, where chemotherapy is followed by bispecific antibodies or the other way around, could potentially minimize the toxicity while maintaining the antitumor efficacy. The combination of bispecific antibodies and chemotherapy continues to evolve, incorporating next-generation antibody formats, such as trispecific and multispecific antibodies, which can simultaneously target multiple pathways and engage more immune cell types, which may demonstrate favorable outcomes. Having said that, establishing more efficacy data from prospective clinical trials on combination treatment with bispecific antibodies and systemic chemotherapy, and then further integrating other therapeutic modalities, such as radiation therapy or immune checkpoint inhibitors, may offer a promising multifaceted strategy.

The role of bsAbs in NSCLC must be evaluated in terms of alternative strategies, such as with ADC and ICI. ADCs like datopotamab deruxtecan deliver cytotoxic agents directly to the tumor cells, resulting in minimal damage to the normal cells. They offer comparable efficacy with potentially fewer immune-related adverse events. Interestingly, ADCs require specific antigen expression, thus limiting their use, while bsAbs can engage immune cells regardless of the immune burden. Likewise, ICIs such as pembrolizumab, atezolizumab, and cemiplimab have transformed NSCLC management, and bsAbs like ivonescimab seek to enhance immune activation through dual targeting. Although bsAbs may help overcome resistance by tempering multiple downstream signaling pathways, ICIs have shown an established efficacy profile and predictive biomarkers. In contrast, bsAbs still require further validation to refine the patient selection. Continued research into this alternative therapeutic approach is crucial to determine the optimal placement of bsAbs in NSCLC treatment.

Despite the encouraging benefits of bispecific antibodies in NSCLC treatment, several limitations obstinate in the currently available studies, including methodological strains, data maturity, real-world applicability, and comparative efficacy. For instance, trials such as PAPILLON and MARIPOSA-2 have varying inclusion criteria and chemotherapy backbones, leading to inconsistencies in interpreting the results. Furthermore, differences in the endpoint and surrogates for long-term outcomes and limited biomarker-driven stratifications make accessing the long-term benefits more challenging. Additionally, a head-to-head comparison of bsAbs with other emerging modalities, including antibody–drug conjugates, ICIs, or novel TKIs, is lacking. Moving on, a limitation to date is that most studies on bispecific antibodies in NSCLC are limited to exploring their potential use as a monotherapy and/or are in early-phase clinical trials, or focus on a specific population cohort, lacking the generalizability of the findings across all NSCLC patients. The existing studies on the combination strategy of bispecific antibodies and chemotherapy are scarce, and often have heterogeneous designs, varying in the chemotherapy selection, dosages, and patient inclusion criteria. Additionally, a significant portion of the data on newer bispecific antibodies may be available only in conference abstracts and preprints, which limits their inclusion and critical evaluation.

As more data become available, advanced combinatorial approaches could position bispecific antibodies at the center of multimodal NSCLC treatment protocols, thereby refining the treatment outcomes. With larger prospective clinical trials, concurrently using bispecific antibodies with chemotherapy may transform the management landscape for advanced NSCLC patients, offering them more effective, durable, and personalized treatment options.

## Figures and Tables

**Table 1:** FDA-approved bispecific antibodies in oncology.

N	Study Phase	Trade Name	Active Ingredient	Target	Year of Approval	Description	Ref.
36	II	Blincyto	Blinatumomab	CD19 and CD3	2014	R/R B-cell ALL, 69% had CR, and 88% achieved MRD. Median OS = 9.8 months (95% CI, 8.5–14.9).	[72]
362	I	Rybrevant	Amivantamab	EGFR and MET	2021	EGFR20 insertion NSCLC, ORR = 40%, mDOR = 11 mo., mPFS = 8.3 mo., TrDR = 13%, treatment discontinuation = 4%.	[15]
378	III	Kimmtrak	Tebentafusp	gp100 and CD3	2022	Metastatic uveal melanoma, 1 year OS = 73% (HR 0.51).	[73]
165	I/II	Tecvayli	Teclistamab	BCMA and CD3	2022	R/R multiple myeloma, ORR = 61.8%, DOR at 9 mo. = 66.5% (95% CI, 38.8%, 83.9%).	[74]
90	1/II	Lunsumio	Mosunetuzumab-axgb	BCMA and CD3	2022	R/R follicular lymphoma, ORR = 80% (95% CI, 70, 88), with 60% achieving CR.	[75]
157	I/II	Epkinly	Epcoritamab-bysp	CD20 and CD3	2023	R/R large B-cell lymphoma, ORR = 63.1%, CR = 38.9% (95% CI, 31.2 to 46.9)	[60]
155	I/II	Columvi	Glofitamab-gxbm	CD20 and CD3	2023	R/R DLBCL, CR = 39% (95% CI, 32 to 48).	[76]
187	I/II	Talvey	Talquetamab-tgvs	GPRC5D and CD3	2023	R/R multiple myeloma, ORR = 73% (95% CI, 63%, 81%).	[77,78]
97	II	Elrexfio	Elranatamab-bcmm	BCMA and CD3	2023	R/R multiple myeloma, ORR = 57.7% (95% CI, 47.3%, 67.7%), DOR at 9 months = 82.3% (95% CI, 67.1%, 90.9%).	[79]
99	II	Imdelltra	Tarlatamab	DLL3 and CD3	2024	Platinum refractory ES-SCLC, ORR = 40% (95% CI, 31, 51), mDOR = 9.7 mo. (range 2.7, 20.7+).	[80]

R/R—relapsed/refractory; ALL—acute lymphocytic leukemia; MRD—molecular residual disease; OS—overall survival, PFS—progression-free survival; DOR—duration of response; TrDR—treatment-related dose reduction; ORR—overall response rate; CR—complete response; HR—hazards ratio; CI—confidence interval; DLBCL—diffuse large B-cell lymphoma; ES-SCLC—extensive-stage small cell lung carcinoma; N—number (of patients); CD—cluster of differentiation; EGFR—epidermal growth factor receptor; BCMA—B-cell maturation antigen; MET—mesenchymal–epithelial transition factor; GPRC5D—G protein-coupled receptor class C group 5 member D; Ref.—reference.

**Table 2:** Ongoing clinical trials utilizing bispecific antibodies with chemotherapy in advanced NSCLC treatment.

NCT	No.	Bispecific Antibody	Target	Chemotherapy Used in Combination	Brief Description/Other Agents Used in Study	Study Start Date	Expected Completion Date
NCT06277674	20	Cadonilimab (AK104)	PD-1/CTLA4	Pemetrexed	Phase II study; NSCLC patients with T790m-negative after TKI resistance; combination of cadonilimab + pemetrexed + anlotinib in subsequent-line setting; outcomes: ORR and PFS.	2 November 2023–	June 2025
NCT05299125	49	Amivantamab	EGFR/MET	Pemetrexed	Phase II; NSCLC with EGFR 19 del and L858R m; combination treatment with amivantamab, lazertinib, and pemetrexed in 1 L setting; outcomes: PFS, ORR, OS, AE, PRO, and compliance.	24 May 2023	April 2028
NCT05215067	40	Cadonilimab (AK104)	PD-1/CTLA4	Docetaxel	Phase II; NSCLC without EGFR- and ALK m; combination treatment with AK104 and docetaxel in 2 L setting; outcomes: ORR, AE, PFS, OS, TTR, DCR, and DOR.	9 March 2022	March 2024
NCT06467500	48	Cadonilimab (AK104)	PD-1/CTLA4	Monotherapy (gemcitabine, pemetrexed, docetaxel, albumin-bound paclitaxel, or vinorelbine)	Phase II; NSCLC patients with driver mutation-negative; 2 L post-ICI failure; outcomes: ORR, DCR, DOR, PFS, OS, and AE.	1 March 2024	30 December 2026
NCT04868877	576	MCLA-129	EGFR/MET	SOC chemotherapy (as per local guidelines)	Phase I/II; NSCLC or other solid tumors; 1 L or refractory; outcomes: MTD, ORR, DOR, DCR, PFS, and AE.	28 April 2021	March 2027
NCT05816499	50	Cadonilimab (AK104)	PD-1/CTLA4	Docetaxel	Phase I/II; ICI and platinum-refractory, driver mutation-ve, and NSCLC; combination treatment with cadonilimab, anlotinib, and docetaxel; outcomes: PFS, ORR, OS, and AE.	16 February 2023	December 2025
NCT04686305	244	Rilvegostomig, Volrustomig	PD-1/TIGIT PD-1/CTLA4	Carboplatin	Phase I; refractory NSCLC, Her2 overexpression +ve, and kinase alteration-ve; multiple arms; combination treatment with T-DXd, volrustomig, and carboplatin (Arm3B), and T-DXd and rilvegostomig with carboplatin (Arm4B); outcomes: AE, DOR, DCR, PFS, and OS.	9 March 2021	December 2025
NCT05812534	36	Cadonilimab (AK104)	PD-1/CTLA4	Carboplatin	Phase II; EGFR- and ALK-ve NSCLC patients with untreated brain metastases; combination treatment with cadonilimab + bevacizumab + carboplatin + pemetrexed; outcomes: ORR, intracranial PFS, PFS, and OS.	December 2023	June 2025
NCT06424821	54	Cadonilimab (AK104)	PD-1/CTLA4	Carboplatin + Paclitaxel (for squamous), Carboplatin + Pemetrexed (for nonsquamous)	Phase II; wild-type EGFR/ALK and PD-L1-ve advanced NSCLC; combination treatment with cadonilimab + platinum-based chemotherapy; outcomes: PFS, OS, ORR, and DOR.	4 July 2023	September 2025
NCT05756972	374	PM8002	PD-L1/VEGF	Carboplatin + Pemetrexed	Phase II/III; EGFR +ve NSCLC, resistant to 1 L TKI; randomized to PM8002 + chemotherapy vs. placebo + chemotherapy; outcomes: ORR, PFS, OS, DCR, DOR, TTR, and AE.	26 June 2023	December 2025
NCT06412471	235	SSGJ-707	PD-1/VEGF	Carboplatin + Paclitaxel (for squamous), Carboplatin + Pemetrexed (for nonsquamous)	Phase II; 1 L NSCLC patients; combination treatment with SSGJ-707 + platinum-based chemotherapy; multiple cohorts; outcomes: ORR, safety and tolerability, and PFS.	26 July 2024	August 2025
NCT05984277	900	Volrustomig (MEDI5752)	PD-1/CTLA4	Carboplatin + Paclitaxel (for squamous), Carboplatin + Pemetrexed (for nonsquamous)	Phase III; EGFR/ALK/ROS1-ve and PD-L1 +ve treatment-naive advanced NSCLC patients; randomized to volrustomig + chemotherapy vs. pembrolizumab + chemotherapy; outcomes: PFS, OS, ORR, and DOR.	24 October 2023	16 May 2029
NCT06173505	168	vudalimab (XmAb20717)	PD-1/CTLA4	Carboplatin + Pemetrexed	Phase I/II; advanced 1 L nonsquamous NSCLC; combination treatment of vudalimab + chemotherapy to evaluate safety in part 1, followed by comparative efficacy with combination of ICI + chemotherapy in part 2; outcomes: phase II dose, PFS, OS, and AE.	27 December 2023	October 2027
NCT04736823	296	Ivonescimab (AK112)	PD-1/VEGF	Carboplatin, Paclitaxel, and Pemetrexed (nonsquamous); Docetaxel	Phase II; advanced NSCLC patients; combination treatment of AK112 with chemotherapy in different cohorts; outcomes: ORR, PFS, and OS.	1 February 2023	March 2025
NCT06196814	150	Ivonescimab (AK112)	PD-1/VEGF	Platinum-based chemotherapy	Phase I/II; EGFR/ROS/ALK +ve NSCLC patients who failed 1 L; combination treatment with AK112 + chemotherapy; outcomes: phase 2 dose, ORR, and PFS.	1 October 2024	1 February 2027
NCT05184712	322	Ivonescimab (AK112 OR SMT112)	PD-1/VEGF	Carboplatin + Pemetrexed	Phase III; multicenter, EGFR +ve, TKI-refractory, nonsquamous, NSCLC patients; randomized to either AK112 + chemotherapy vs. placebo + chemotherapy; outcomes: PFS, OS, ORR, DCR, DOR, TTR, and AE.	25 January 2022	2 November 2025
NCT05904379	233	Ivonescimab (AK112) + Cadonilimab (AK104)	PD-1/VEGF & PD-1/CTLA4	Carboplatin, Paclitaxel, and Pemetrexed (nonsquamous); Docetaxel	Phase I/II; EGFR/ALK-ve, advanced NSCLC; combination treatment with either AK112 + AK104 or AK112 + AK104 + chemotherapy in different cohorts; outcomes: AE, ORR, OS, DOR, DCR, and PFS.	13 July 2023	January 2027
NCT05840016	396	Ivonescimab (AK112)	PD-1/VEGF	Carboplatin + Paclitaxel	Phase III; advanced squamous NSCLC, driver mutation-ve; evaluating the efficacy and safety of AK112 + chemotherapy vs. tislelizumab + chemotherapy; outcomes: PFS, OS, ORR, DOR, DCR, DCR, TTR, and AE.	17 Aug 2023	Decemeber 2025
NCT06357533	675	Rilvegostomig	PD-1/TIGIT	Cytotoxic payload (deruxtecan) of Datopotamab Deruxtecan	Phase III; advanced nonsquamous NSCLC, driver mutation-ve, PDL1 > 50%; evaluating rilvegostomig monotherapy or the combination of datopotamab deruxtecan versus pembrolizumab; outcomes: PFS, OS, and ORR.	11 April 2024	24 May 2030

OS—overall survival, PFS—progression-free survival; DOR—duration of response; ORR—overall response rate; CR—complete response; DCR—disease control rate; No.—number (of patients); EGFR—epidermal growth factor receptor; VEGF—vascular endothelial growth factor; MET—mesenchymal–epithelial transition factor; ALK—anaplastic lymphoma kinase; TIGIT—T-cell immunoreceptor with Ig and ITIM domains; AE—adverse event; TKI—tyrosine kinase inhibitor; PD-1/PD-L1—programmed death 1/programmed death ligand 1; CTLA4—cytotoxic T-lymphocyte-associated antigen 4; NCT—national clinical trial.

**Table 3:** PD-1- or PD-L1-targeted bispecific antibodies in NSCLC.

PD-1- or PD-L1-Targeted Bispecific Antibodies	Target Identified	NCT
Rilvegostomig (AZD2936)	PD-1/TIGIT	NCT06357533, NCT06627647, NCT06564844, NCT04995523, NCT04612751
HLX301	PD-L1/TIGIT	NCT05102214
Acasunlima (GEN1046)	PD-L1/4-1BB	NCT06635824, NCT05117242
IBI318	PD-1/PD-L1	NCT04777084
PF-07257876	PD-L1/CD47	NCT04881045
IMM2520	PD-L1/CD47	NCT05780307
Lomvastomig	PD-1/TIM-3	NCT03708328
AZD7789	PD-1/TIM-3	NCT04931654
IBI363	PD-L1/IL-2α	NCT06468098, NCT06081907
RO7247669	PD-1/LAG-3	NCT04140500
MGD013	PD-1/LAG-3	NCT03219268
CDX-585	PD-L1/ILT4	NCT05788484
XmAb23104 (XmAb 104)	PD-1/CD278	NCT03752398
SHR-1701	PD-L1/TGF-βRII	NCT05177497, NCT04937972
HB0025	PD-L1/VEGF	NCT04678908

LAG—lymphocyte-activation gene 3; TIGIT—T-cell immunoreceptor with Ig and ITIM domains; PD-1/PD-L1—programmed death 1/programmed death ligand 1; NCT—national clinical trial; CD—cluster of differentiation; TIM 3—T-cell immunoglobulin and mucin domain-containing protein 3.

## Abbreviations

NSCLC	non-small-cell lung cancer
TME	tumor microenvironment
BsAbs	bispecific antibodies
